# Optimization of Kidney Disease: Improving Global Outcomes Criteria for AKI for Pediatric Population

**DOI:** 10.1016/j.ekir.2025.11.026

**Published:** 2025-11-26

**Authors:** Chao Zhang, Ruohua Yan, Xiaohang Liu, Xiaolu Nie, Yaguang Peng, Nan Zhou, Xiaoxia Peng

**Affiliations:** 1 Center for Clinical Epidemiology and Evidence-based Medicine, National Center for Children Health, Beijing Children’s Hospital, Capital Medical University, Beijing, China; 2 Department of Nephrology, National Center for Children Health, Beijing Children’s Hospital, Capital Medical University, Beijing, China

**Keywords:** acute kidney injury, KDIGO, pediatric

## Abstract

**Introduction:**

Accurate detection and staging of acute kidney injury (AKI) is important in clinical practice to aid timely management. The main purpose of this study is to establish a pediatric version of Kidney Disease: Improving Global Outcomes (KDIGO, pKDIGO) criteria for pediatric population.

**Methods:**

The pKDIGO criteria defined AKI following the principles of KDIGO, in which the threshold of absolute increase in serum creatinine (SCr) or absolute decrease in estimated glomerular filtration rate (GFR, eGFR) to diagnose AKI has been revised to eliminate the impacts of age and sex of children. Then, AKI defined by pKDIGO were compared with that defined by KDIGO, modified KDIGO (mKDIGO), pediatric reference change value optimized for AKI in children (pROCK), and pediatric Risk for renal dysfunction, Injury to the kidney, Failure of kidney function, Loss of kidney function, and End-stage renal disease (RIFLE, pRIFLE) based on 2 retrospective cohorts in China: Beijing Children’s Hospital (BCH) cohort and intensive care units (ICUs) of the Children’s Hospital of Zhejiang University School of Medicine (ICU) cohort. The performance of different AKI definitions was compared based on the area under the receiver operating characteristic curves (AUCs) for predicting the in-hospital death.

**Results:**

Total of 57,229 children in the BCH cohort and 8276 children in the ICU cohort were used to evaluate the performance of pKDIGO. In the BCH cohort, AUCs for predicting mortality by AKI defined based on pKDIGO (AUC = 0.75, 0.72–0.78) were higher than that defined by other definitions. The risk of death increases with higher stage of AKI defined by pKDIGO. Similar results were observed in the ICU cohort.

**Conclusion:**

The pKDIGO criteria showed a better ability to identify patients with AKI and predict in-hospital death in children, both in general wards and ICUs.

## Graphical abstract

**Figure:**
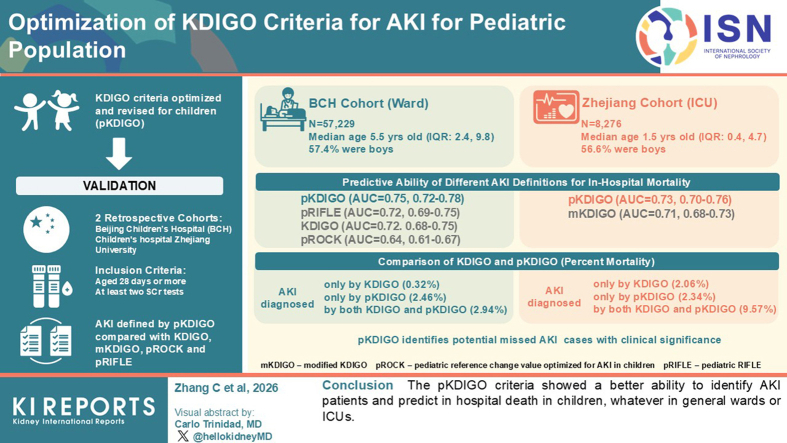

AKI is an adverse event characterized by a rapid decrease in renal excretory function, occurring in approximately 5% to 26.9% of hospitalized children.^1^^,^^2^ It has been reported that AKI is associated with poor prognosis in pediatric patients, including the length of hospital stay, chronic kidney disease, and mortality.^3^, ^4^, ^5^ Therefore, timely and accurate diagnosis of AKI is of great significance for the improvement of clinical decision-making and patients’ prognosis. To harmonize the previously proposed AKI criteria, including RIFLE,^6^ pRIFLE^7^ and Acute Kidney Injury Network,^8^ the KDIGO Work Group proposed to establish a common definition, that is, an updated consensus definition of AKI in 2012 (Table 1).^9^ To date, the KDIGO AKI definition is the most widely used in both adult and pediatric populations.^10^^,^^11^

**Table 1:** KDIGO and pKDIGO criteria

AKI criteria	Stage	SCr	eGFR	Urine output
KDIGO	1	Increase 50%–99%^b^ from baseline or increase of 0.3 mg/dl^a^		<0.5 ml/kg/h for 6 h
	2	Increase 100%–199%^b^ from baseline		<0.5 ml/kg/h for 12 h
	3	Increase ≥ 200%^b^ from baseline or SCr ≥ 4 mg/dl	eGFR <35 ml/min per 1.73 m^2^	<0.3 ml/kg/h for 24 h or anuria for 12 h
pKDIGO	1	Increase 50%–99%^b^ from baseline or increase of 0.3×Q_age_/0.859 mg/dl^a^		<0.5 ml/kg/h for 6 h
	2	Increase 100–199%^b^ from baseline		<0.5 ml/kg/h for 12 h
	3	Increase ≥ 200%^b^ from baseline or SCr ≥ 4 ×Q_age_/0.859 mg/dl	eGFR <35×(1-e^-age/0.5^) ml/min per 1.73 m^2^	<0.3 ml/kg/h for 24 h or anuria for 12 h

eGFR, estimated glomerular filtration rate; KDIGO, Kidney Diseases: Improving Global Outcomes; pKDIGO, pediatric version of KDIGO; SCr, serum creatinine.

Q_age_=0.21 + 0.057 × Age - 0.0075×Age^2^+ 0.00064 × Age^3^- 0.000016 × Age^4^ for boys.

Q_age_=0.23 + 0.034 × Age - 0.0018×Age^2^+ 0.00017× Age^3^- 0.0000051 × Age^4^ for girls.

a increase within 48 h.

b increase within 7 d.

Because the KDIGO definition was originally developed for adults, there are still doubts regarding the applicability of the KDIGO AKI definition among children.^12^ For example, the KDIGO defines an increase of 0.3 mg/dl in SCr within 48 hours or an increase of 50% to 99% from baseline in SCr within 7 days as stage 1 AKI. However, with particular reference to children, the concentration of SCr increases continuously with age until adulthood. Especially before the age of 6 years, the concentration of SCr in children is much lower than that in adults.^13^ Given that the average SCr level varies with age, curious results are obtained in some situations. For example, a boy aged between 1 and 2 years with a baseline SCr of 0.3 mg/dl (reference interval in China: 0.2–0.4 mg/dl), an increase of 0.3 mg/dl in absolute SCr means an increase of 100% from baseline, which already fulfills the criteria of stage 2 AKI (an increase of 100%–199% from baseline). Another controversy is that the KDIGO defines an eGFR < 35 ml/min per 1.73 m^2^ as stage 3 AKI for patients aged < 18 years, whereas the developmental curve of GFR is reflected as a rapid increase from birth, approaching adult levels at the age of 2 years.^14^ Therefore, eGFR criteria are not applicable to younger children.

In this way, some studies have adapted the KDIGO definition for the pediatric population. One modification was applying the eGFR < 35 ml/min per 1.73 m^2^ criteria only to children aged > 3 months.^15^ Another modification suggested an SCr^3^ 2.5 mg/dl (rather than 4.0 mg/dl) as the threshold for AKI stage 3 for neonates.^16^ However, these revisions were made only for children with younger age. Given that SCr and eGFR levels change continuously across the entire age spectrum for the pediatric population, the above revisions may still be unreasonable, especially for school-age children and adolescents.

Therefore, this study aimed to establish a pKDIGO definition using age-specific SCr or eGFR rather than a fixed bound of SCr or eGFR across the entire age spectrum to define AKI in the pediatric population.

## Methods

The present study was performed in accordance with the recommendations laid out in the World Medical Association Declaration of Helsinki. Ethical approval was obtained from the Institutional Review Board of BCH, Capital Medical University (IRB No. [2022]-E-207-Y).

### Development of the pKDIGO Criteria

#### Age Dependency of eGFR and SCr

In healthy children, the GFR value depends on the maturity of kidney development. Therefore, GFR generally increases after birth and reaches the adult level around 107.3 ,ml/min per 1.73 m^2^ at about 2 years, and then remains stable.^14^ Pottel *et al.*^17^^,^^18^ showed the relationship between age and median eGFR (i.e. *M*) in a healthy pediatric population using the following formula:

$$M=107.3\left[1-e^{-Age/0.5}\right]\;\text{ml}/\min\text{per}1.73\;m^{2}$$

SCr is a typical endogenous kidney biomarker that can be filtered freely by the glomerulus. Because the concentration of SCr in the human body is affected by muscle mass, during childhood, SCr continuously increases with age and presents a sex difference after puberty, with SCr of boys being slightly higher than SCr of girls.^19^^,^^20^ Pottel *et al.* used 2 polynomials to fit the median SCr (i.e. *Q*) by age (in years) for healthy girls and boys^17^^,^^18^:

$$Q=0.21+0.057\times Age-0.0075\times {Age}^{2}+0.00064\times {Age}^{3}-0.000016\times {Age}^{4}\;\left(\text{boys}\right)$$

$$Q=0.23+0.034\times Age-0.0018\times {Age}^{2}+0.00017\times {Age}^{3}-0.0000051\times {Age}^{4}\;\left(\text{girls}\right)$$

In Supplementary Figure S1, we show the age-dependent trend of median eGFR (*M*) and median SCr (*Q*) in healthy children. The applicability of the standard eGFR and SCr has been validated in healthy children in many countries and regions.^13^^,^^21^

#### Modification of the KDIGO Criteria

To develop the pKDIGO criteria, we first assumed that the median SCr for boys aged 18 years was close to the adult level. In this way, the SCr threshold for defining AKI in boys aged 18 years should be the same as that in the KDIGO criteria. Considering the correlation of SCr with sex and age, the SCr threshold for AKI in children aged < 18 years old or girls should be proportionally lowered. Taking the median SCr (*Q*) of boys at age 18 years (0.859 mg/dl) as a benchmark value (Supplementary Figure S1), the SCr threshold in KDIGO to diagnose AKI in children aged < 18 years was revised into the SCr threshold multiplying a ratio, that is, Q_age,sex_ (median SCr at corresponding age and sex) divided by 0.859. In the same way, the cutoff point (35 ml/min per 1.73 m^2^) of eGFR of children was modified to 35×(1-e^-age/0.5^). As summarized in Table 1, the KDIGO criteria has been optimized in the following issues to improve its applicability in children:

1. An increase of 0.3 mg/dl in SCr within 48 hours is replaced by an increase of 0.3×Q_age,sex_/0.859 mg/dl for stage 1 AKI;
2. A current SCr > 4 mg/dl is replaced by 4×Q_age,sex_ /0.859 mg/dl for stage 3 AKI;
3. A current eGFR < 35 ml/min per 1.73 m^2^ is replaced by 35×M_age_/107.3 ml/min per 1.73 m^2^, that is, 35×(1-e^-age/0.5^) ml/min per 1.73 m^2^ for stage 3.

In Supplementary Figure S2 to S4, we plotted the distribution of relative changes of Scr and eGFR (calculated based on the data in healthy Chinese children^19^) and absolute changes (the modified AKI criteria defined by pKDIGO) across the pediatric age spectrum for visualization.

### Validation of the pKDIGO Criteria

#### Study Population

Two retrospective cohorts from China were used to validate the pKDIGO criteria. The BCH cohort consisted of all patients admitted to the general wards in BCH, Capital Medical University from 2015 to 2023. The ICU cohort consisted of all patients admitted to the ICU of the Children’s Hospital of Zhejiang University School of Medicine from 2010 to 2019. The inclusion criteria were defined as follows: (i) aged ≥ 28 days; (ii) with ≥ 2 SCr tests. The exclusion criterion was being diagnosed as chronic kidney disease at admission. The same eligibility criteria were used for the 2 cohorts.

#### The Definitions of AKI

In both cohorts, demographics, laboratory testing data, comorbidities, and death records were extracted from electronic medical records. The baseline SCr was selected based on a dynamic method (Supplementary Figure S5). First, the values of SCr during hospitalization were sorted sequentially in the order of test time. For any time point when AKI was defined, the baseline SCr was dynamically defined as the average SCr value over the 7 days prior to this time point. Then, each available SCr value over the 7 days following that time point was compared to that baseline mean SCr.^22^^,^^23^ eGFR was estimated by the full age spectrum equation based on SCr, sex, and age, that is, eGFR = *M*/(SCr [mg/dl]/*Q*).^17^ Considering that urine output records were unavailable in the BCH cohort, all AKI definitions did not take into account the influence of urine output in the primary analysis.

In the first sensitivity analysis, eGFR was estimated using the Schwartz equation based on SCr and height, that is, eGFR = 0.413′height (cm)/Scr (mg/dl).^24^ Because data on height were unavailable in the ICU cohort, this analysis was only conducted in the BCH cohort. In the second sensitivity analysis, AKI was reidentified by considering urine output. This analysis was only conducted in the ICU cohort.

#### The Performance Evaluation of pKDIGO

AKI defined by pKDIGO were compared with that defined by KDIGO, mKDIGO,^25^ (pROCK),^12^ and pRIFLE^7^, respectively, based on BCH cohort and ICU cohort. Details of the definitions of AKI mentioned above are presented in Supplementary Table S1.

Owing to the lack of a gold standard for diagnosing AKI, the predictive ability of AKI diagnosed using different definitions for predicting in-hospital death was evaluated. The better predictive ability indicated a better diagnostic performance. In addition, Kaplan–Meier survival curves were drawn to describe the impacts of different AKI stages defined by KDIGO, pKDIGO, and pRIFLE on mortality.

### Statistical Analysis

Data preprocessing was conducted using SAS, version 9.4 (SAS Institute Inc, Cary, NC). Python software (version 3.10.4) and R software (version 4.1.0) were used to plot the figures and for statistical analysis. The predictive ability of AKI based on different definitions to predict in-hospital death was compared using the AUC. In addition, the multivariate logistic regression analysis was used to estimate the association between AKI and in-hospital death after adjusting for confounders, including age and sex.

## Results

### Characteristics of the Participants

According to the eligibility criteria, 57,229 of 377,843 children were selected based on the BCH cohort (Supplementary Figure S6a), in which 7457 (13.0%), 36,071 (63.0%), and 13,701 (24.0%) were in infancy (28 days to < 1 year), childhood (1 to < 10 years), and adolescence (10 to < 18 years), respectively. The median age was 5.5 (interquartile range: 2.4–9.8) years old and 32,851 (57.4%) of the children were boys (Table 2).

**Table 2:** The demographic and clinical characteristics of participants

	BCH cohort (*N* = 57,229)	ICU cohort (*N* = 8276)
Sex (Male), *n* (%)	32851 (57.4)	818 (56.6)
Age (yrs), Median (IQR)	5.5 (2.4–9.8)	1.5 (0.4–4.7)
Infancy, *n* (%)	7457 (13.0)	3436 (41.5)
Childhood, *n* (%)	36,071 (63.0)	4097 (49.5)
Adolescence, *n* (%)	13,701 (24.0)	743 (9.0)
Death, *n* (%)	259 (0.5)	359 (4.3)
LOS, Median (IQR)	11 (7–17)	13 (8–20)

BCH, Beijing Children’s Hospital; ICU, intensive care unit; IQR, interquartile range; LOS, length of stay.

Simultaneously, 8276 cases were filtered from 13,449 inpatients in the ICU cohort (Supplementary Figure S6b). In the ICU cohort, 3436 (41.5%), 4097 (49.5%), and 743 (9.0%) patients were in infancy, childhood, and adolescence, respectively. The median age was 1.5 (interquartile range: 0.4, 4.7) years, and 818 (56.6%) of the children were boys (Table 2).

### AKI Cases With Different Definitions

**Figure 1:**
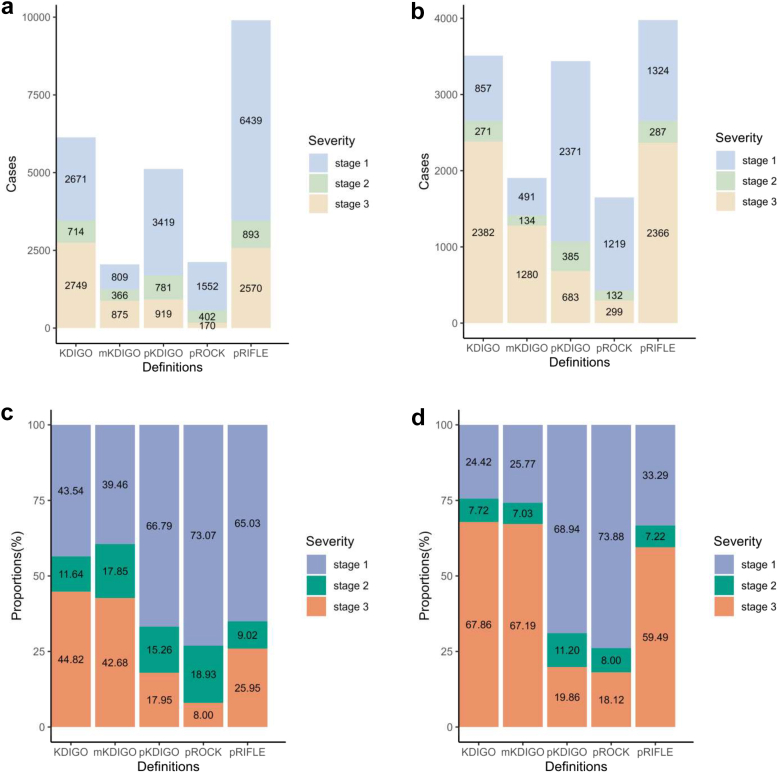
Histogram of acute kidney injury stages distribution. (a) Number of cases in the Beijing Children’s Hospital cohort. (b) Number of cases in the intensive care unit cohort. (c) Proportions in the Beijing Children’s Hospital cohort. (d) Proportions in the intensive care unit cohort. KDIGO, Kidney Diseases: Improving Global Outcomes; mKDIGO, modified KDIGO; pKDIGO, pediatric version of KDIGO; pRIFLE, pediatric Risk for renal dysfunction, Injury to the kidney, Failure of kidney function, Loss of kidney function, and End-stage renal disease; pROCK, pediatric reference change value optimized for AKI in children.

In the BCH cohort, the number of AKI cases diagnosed using KDIGO, mKDIGO, pKDIGO, pROCK, and pRIFLE were 6134, 2050, 5119, 2124, and 9902, respectively. In the ICU cohort, the number AKI cases diagnosed using KDIGO, mKDIGO, pKDIGO, pROCK, and pRIFLE were 3510, 1905, 3439, 1650, and 3977, respectively. However, in the BCH cohort or in ICU cohort, most number of patients with AKI were identified using the pRIFLE; whereas, mKDIGO and pROCK identified the least number of patients with AKI (Figure 1). In the BCH cohort, the proportions of patients in the third stage of AKI among all AKI cases according to KDIGO, mKDIGO, pKDIGO, pROCK, and pRIFLE were 44.82%, 42.68%, 17.95%, 8.00%, and 25.95%, respectively (Figure 1c). In the ICU cohort, the corresponding proportions were 67.86%, 67.19%, 19.86%, 18.12%, and 59.49%, respectively (Figure 1d). As illustrated in Figure 1c and d, a large proportion of patients were identified as stage 3 AKI, which is not in line with the convention, especially in the ICU cohort.

**Figure 2:**
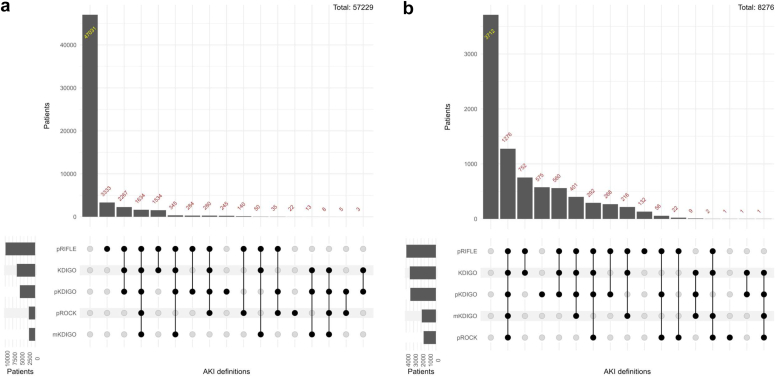
Upset plot of AKI definitions. (a) Beijing Children’s Hospital cohort. (b) Intensive care unit cohort. AKI, acute kidney injury; KDIGO, Kidney Diseases: Improving Global Outcomes; mKDIGO, modified KDIGO; pKDIGO, pediatric version of KDIGO; pRIFLE, pediatric Risk for renal dysfunction, Injury to the kidney, Failure of kidney function, Loss of kidney function, and End-stage renal disease; pROCK, pediatric reference change value optimized for AKI in children.

Upset plot further shows the overlap of AKI cases detected across the 5 definitions of AKI (Figure 2). In the BCH cohort, 3333 of 57,299 of patients (5.82%) with AKI were identified only by pRIFLE, 245 of 57,299 (0.43%) were identified only by pKDIGO, and 22 of 57,299 (0.04%) were identified only by pROCK (Figure 2a). In the ICU cohort, 575 of 8276 patients (6.95%) with AKI were identified only by pKDIGO, 132 of 8276 (1.59%) were identified only by pRIFLE, and 1 of 8276 (0.01%) were identified only by pROCK (Figure 2b). This indicated that pKDIGO, pROCK, and pRIFLE might be more sensitive for detecting AKI.

### In-Hospital Death of Patients With AKI With Different Definitions

As shown in Supplementary Table S2, the in-hospital mortality rates in the BCH cohort and the ICU cohort were 0.45% and 4.34%, respectively. However, the risk of mortality was increased in hospitalized patients with AKI than in those without AKI, regardless of AKI definitions; whether in the BCH cohort (KDIGO, 2.27%; mKDIGO, 3.80%; pKDIGO, 2.89%; pROCK, 3.91%; pRIFLE, 1.56%; vs. 0.45%) or in ICU cohort (KDIGO, 7.49%; mKDIGO, 11.44%; pKDIGO, 7.68%; pROCK, 11.15%; pRIFLE, 6.94%; vs. 4.34%) (Supplementary Table S2).

What is striking is that potentially missed AKI cases with clinical significance were identified using pKDIGO (Supplementary Table S2). For example, the number of AKI cases identified using only pKDIGO (*n* = 245 in BCH cohort) was far less than that of only pRIFLE (*n* = 3333 in BCH cohort); however, the mortality of AKI identified using only pKDIGO (3.67%) was higher than of pRIFLE (0.27%). In the ICU cohort, the number of AKI cases identified using only pKDIGO (*n* = 575) was more that of only pRIFLE (*n* = 132), and the mortality of AKI identified using only pKDIGO (1.22%) was also higher than that of pRIFLE (0.76%).

The association between mortality and AKI identified using different AKI definitions was evaluated using logistic regression models after adjusting for confounders of age and sex (Table 3). In the BCH cohort, the risk of death in patients identified as stage 2 AKI was higher than that in patients with stage 3 AKI when they were identified according to KDIGO, pROCK, and pRIFLE, which seems theoretically anomalous. In contrast, the risk of death increased with severity of AKI stage in patients with AKI identified using pKDIGO and mKDIGO, showing a dose-response relationship.

**Table 3:** Association of mortality with AKI identified by different AKI definitions

Definitions	BCH cohort		ICU cohort	
	Mortality *n* (%)	OR (95% CI)^a^	Mortality *n* (%)	OR (95% CI)^a^
KDIGO				
Non-AKI	120/51,095 (0.23)	NA	96/4766 (2.01)	NA
Any stages	139/6134 (2.27)	9.28 (7.21–11.97)	263/3510 (7.49)	4.40 (3.45–5.65)
Stage 1	56/2671 (2.10)	8.88 (6.41–12.17)	46/857 (5.37)	2.73 (1.89–3.88)
Stage 2	27/714 (3.78)	16.24 (10.41–24.44)	15/271 (5.54)	2.94 (1.61–5.01)
Stage 3	56/2749 (2.04)	7.75 (5.42–10.99)	202/2382 (8.48)	5.61 (4.29–7.39)
mKDIGO				
Non-AKI	181/55,179 (0.33)	NA	141/6371 (2.21)	NA
Any stages	78/2050 (3.80)	14.26 (10.79–18.68)	218/1905 (11.44)	5.69 (4.58–7.10)
Stage 1	18/809 (2.22)	9.75 (5.68–15.79)	34/491 (6.92)	3.15 (2.09–4.63)
Stage 2	13/366 (3.55)	13.59 (7.26–23.33)	9/134 (6.72)	3.07 (1.42–5.83)
Stage 3	47/875 (5.37)	17.03 (12.12–23.48)	175/1280 (13.67)	7.30 (5.77–9.25)
pKDIGO				
Non-AKI	111/52,110 (0.21)	NA	95/4837 (1.96)	NA
Any stages	148/5119 (2.89)	13.49 (10.54–17.32)	264/3439 (7.68)	4.21 (3.32–5.37)
Stage 1	74/3419 (2.16)	9.98 (7.39–13.40)	88/2371 (3.71)	1.99 (1.48–2.67)
Stage 2	31/781 (3.97)	18.47 (12.11–27.37)	23/385 (5.97)	3.26 (1.99–5.14)
Stage 3	43/919 (4.68)	23.19 (16.03–32.95)	153/683 (22.40)	14.41 (11.00–18.96)
pROCK				
Non-AKI	176/55,105 (0.32)	NA	175/6626 (2.64)	NA
Any stages	83/2124 (3.91)	15.5 (11.78–20.24)	184/1650 (11.15)	4.62 (3.73–5.73)
Stage 1	46/1552 (2.96)	11.39 (8.08–15.75)	94/1219 (7.71)	3.08 (2.37–3.98)
Stage 2	29/402 (7.21)	30.43 (19.75–45.40)	49/132 (37.12)	21.98 (14.87–32.24)
Stage 3	8/170 (4.71)	19.87 (8.78–38.94)	16/299 (5.57)	5.83 (3.99–8.36)
pRIFLE				
Non-AKI	105/47,327 (0.22)	NA	83/4299 (1.93)	NA
Any stages	154/9902 (1.56)	6.65 (5.17–8.59)	276/3977 (6.94)	4.10 (3.19–5.32)
Stage 1	71/6439 (1.10)	4.86 (3.58–6.57)	59/1324 (4.46)	2.38 (1.68–3.33)
Stage 2	35/893 (3.92)	17.83 (11.93–26.04)	16/287 (5.57)	3.10 (1.72–5.25)
Stage 3	48/2570 (1.87)	7.25 (4.90–10.60)	201/2366 (8.50)	5.84 (4.41–7.81)

AKI, acute kidney injury; BCH, Beijing Children’s Hospital; CI, confidence interval; ICU, intensive care unit; KDIGO, Kidney Diseases: Improving Global Outcomes; mKDIGO, modified KDIGO; NA, not available; OR, odds ratio; pKDIGO, pediatric version of KDIGO; pRIFLE, pediatric Risk for renal dysfunction, Injury to the kidney, Failure of kidney function, Loss of kidney function, and End-stage renal disease; pROCK, pediatric reference change value optimized for AKI in children.

Odds ratios are adjusted for age and sex.

a Logistic regression analysis with non-AKI as reference.

### Predictive Ability of Different AKI Definitions for In-Hospital Outcomes

**Figure 3:**
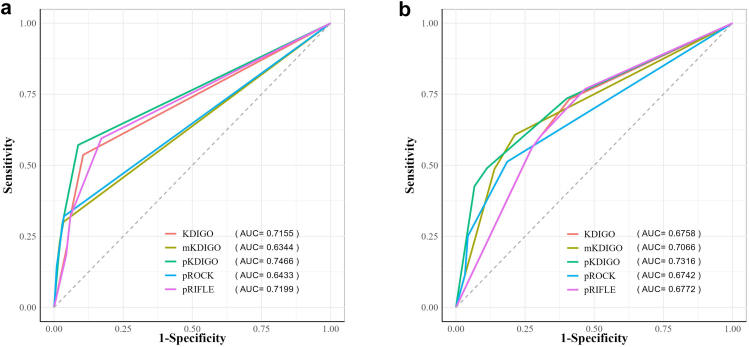
Predictive ability of acute kidney injury definitions to predict in-hospital death. (a) Beijing Children’s Hospital cohort. (b) Intensive care unit cohort. KDIGO, Kidney Diseases Improving Global Outcomes; mKDIGO, modified KDIGO; pKDIGO, pediatric version of KDIGO; pRIFLE, pediatric Risk for renal dysfunction, Injury to the kidney, Failure of kidney function, Loss of kidney function, and End-stage renal disease; pROCK, pediatric reference change value optimized for AKI in children.

As shown in Figure 3, pKDIGO outperformed other definitions in predicting in-hospital death among the general inpatients of BCH cohort (AUC = 0.75, 0.72–0.78). Following pKDIGO were pRIFLE (AUC = 0.72, 0.69–0.75) and KDIGO (AUC = 0.72, 0.68–0.75) (Figure 3a). In the ICU cohort, pKDIGO had the best performance (AUC = 0.73, 0.70–0.76). mKDIGO ranked after pKDIGO (AUC = 0.71, 0.68-0.73) (Figure 3b).

In the sensitivity analysis, 20,869 patients who had data on height in the BCH cohort were analyzed to examine the impacts of different eGFR equations on AKI definitions. In Supplementary Figure S2, we show the predictive performance of AKI when full age spectrum equation (Supplementary Figure S7a) and Schwartz equation (Supplementary Figure S7b) were used to identify AKI. The AUCs were similar for all AKI definitions. Then, to examine the influence of urine output on AKI definition, AKI in the ICU cohort was reidentified by taking into account the urine output (Supplementary Figure S8). In Supplementary Figure S8a, we show the receiver operating characteristic curve of AKI cases when the urine output was missed; and in Supplementary Figure S8b, we show the receiver operating characteristic curve of AKI cases when the urine output was used for AKI definition. In Supplementary Figure S8, we show few difference that could affect the overall results.

### Comparison of KDIGO and pKDIGO

**Figure 4:**
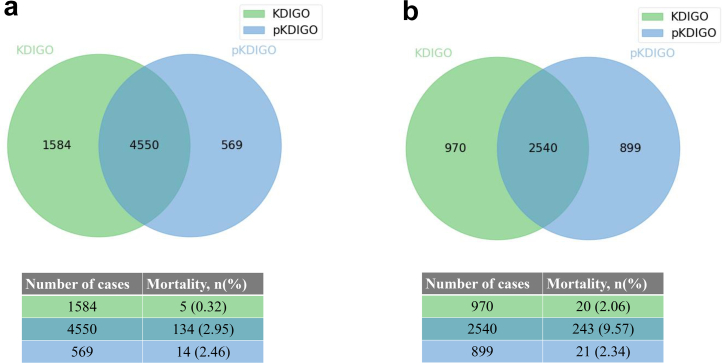
Venn diagram of KDIGO versus pKDIGO definition with mortality. (a) Beijing Children’s Hospital cohort. (b) Intensive care units cohort.

Given that the pKDIGO is a modified version of KDIGO, they may have similar characteristics. We used a Venn diagram to demonstrate their ability to detect and stage AKI (Figure 4). In Figure 4a, we demonstrate the overlap between AKI cases identified using KDIGO and those of pKDIGO, as well as the mortality of each subset in the BCH cohort. As shown in Figure 4a, 4550 patients were defined as having AKI using both KDIGO and pKDIGO simultaneously, with a mortality rate of 2.95% (134/4550). Among the 1584 AKI cases diagnosed using only KDIGO, 20 (0.32%) died in the hospital; among the 569 AKI cases diagnosed using only pKDIGO, 14 (2.46%) died in the hospital. In Figure 4b (ICU cohort), 2540 patients were defined as having AKI using both KDIGO and pKDIGO simultaneously, with a mortality rate of 9.57% (243/2540). Among the 970 AKI cases diagnosed using only KDIGO, 20 (2.06%) died in the hospital; among 899 AKI cases diagnosed using only pKDIGO, 21 (2.34%) died in the hospital.

**Figure 5:**
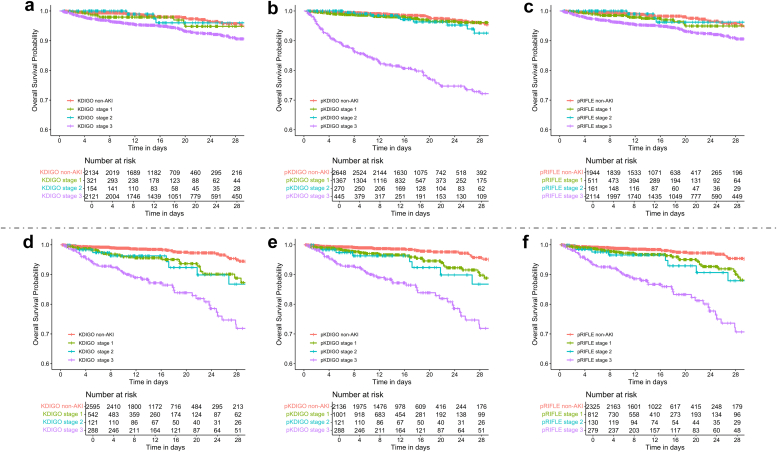
The survival curves of different acute kidney injury stages diagnosed using KDIGO versus pKDIGO versus pRIFLE in the intensive care unit cohort. (a) KDIGO (children aged ≤ 2 years in the intensive care unit cohort). (b) pKDIGO (children aged ≤ 2 years in the intensive care unit cohort). (c) pRIFLE (children aged ≤ 2 years in the intensive care unit cohort). (d) KDIGO (children aged > 2 years in the intensive care unit cohort cohort). (e) pKDIGO (children aged > 2 years in the intensive care unit cohort cohort). (f) pRIFLE (children aged > 2 years in the intensive care unit cohort cohort). AKI, acute kidney injury; KDIGO, Kidney Diseases: Improving Global Outcomes; pKDIGO, pediatric version of KDIGO; pRIFLE, pediatric Risk for renal dysfunction, Injury to the kidney, Failure of kidney function, Loss of kidney function, and End-stage renal disease.

As for the AKI stages identified using KDIGO and pKDIGO, the results are shown in Supplementary Figure S9 (BCH cohort) and Supplementary Figure S10 (ICU cohort), both showing similar results. From the survival curves (Figure 5), it can be observed that pKDIGO did better on mortality risk stratification than KDIGO and pRIFLE in ICU cohort, especially for children aged < 2 years.

## Discussion

In the present study, the pKDIGO criteria defined AKI following the principles of KDIGO, in which the threshold of absolute increase in Scr or absolute decrease in eGFR to diagnose AKI has been revised to eliminate the impacts of age and sex of children.

The KDIGO definition has been validated in several studies on adults and children. Although many studies have noted that KDIGO is not suitable for pediatric patients and modified it for a specific population, none of them have fully accounted for the trend of eGFR and SCr by age and adapted it across all age spectra of children.^15^^,^^16^ We combined the median SCr and eGFR values proposed by Pottel *et al.* and the KDIGO criteria and used 2 retrospective cohorts to validate the newly proposed pKDIGO. A major strength of pKDIGO is that it preserves the framework of the classical KDIGO definition and shows superiority in predicting in-hospital outcomes.

In the BCH cohort, pKDIGO showed the best performance in predicting in-hospital mortality. Compared with pKDIGO, KDIGO and pRIFLE had lower AUC, followed by mKDIGO and pKDIGO. This might be attributed to the lower number of cases detected by mKDIGO and pROCK, which missed some patients with AKI with a high risk of death. In comparison, KDIGO and pRIFLE identified a greater number of patients with AKI,^26^, ^27^, ^28^ as well as pKDIGO. In addition to the AUC, pKDIGO showed the strongest detection ability for AKI among all definitions. Among 245 patients with AKI detected only using pKDIGO, 9 (3.67%) died in the hospital. This led us to believe that pKDIGO had the ability to detect some high-risk patients missed by other definitions. The odds ratio calculated using the multivariate logistic model showed that more severe AKI stages defined by pKDIGO had a higher adjusted odds ratio for mortality. In contrast, stage 3 AKI had a lower odds ratio than stage 2 AKI in the KDIGO and pRIFLE groups. The anomalous result was presumably because the fact that most patients at lower age were determined as AKI stage 3 using KDIGO and pRIFLE, but they were actually not AKI cases. The Venn diagrams and survival curves for different age subgroups may serve as corroborating evidence (Supplementary Figures S11–S13 and Figure 5). Logistic regression showed that stage 2 AKI detected by pROCK had a higher odds ratio than stage 3 AKI, which was consistent with a previous study.^29^

In the ICU cohort, pKDIGO performed the best in predicting in-hospital death, followed by mKDIGO. The predictive ability of pKDIGO is stable in both general wards and ICUs, which may be because the advantages of the age-dependent criteria. Although we used the median eGFR and SCr proposed for Belgian children, previous studies have demonstrated no racial differences in SCr levels between Caucasian and East Asian pediatric populations,^20^ meaning that our work can be generalized to different races.

Currently, the bedside Schwartz equation is the most widely used SCr-based method for estimating GFR in children. However, this equation was derived from children with established kidney disease and notable growth retardation.^21^^,^^30^ Thus, many authors have questioned the applicability of this equation to children without kidney disease.^31^ In this study, we compared the predictive ability of AKI definitions based on full age spectrum equation and Schwartz equation. The results showed that full age spectrum equation and Schwartz equation were almost equivalent in identifying AKI and predicting in-hospital mortality. However, the AUC was slightly increased for pRIFLE when Schwartz equation was used to define AKI, which may be attributed to the fact that the pRIFLE definition was originally derived using Schwartz equation.^6^

There are some limitations of this study. First, pKDIGO has only been validated by predicting in-hospital mortality rates in pediatric patients. In fact, the in-hospital mortality rates of the BCH and ICU cohorts were 0.45% and 4.34%, respectively, which is unbalanced. Therefore, the AUC for predicting mortality by AKI diagnosed based on pKDIGO is still moderate, although better than other AKI criteria. As is well-known, timely and accurate detection of AKI may directly affect long-term kidney function. Unfortunately, there was no follow-up on long-term kidney function in these 2 retrospective cohorts. We hope that pKDIGO can attract the attention of peers, so that in the future, the impact of pKDIGO on long-term renal function can be evaluated based on prospective cohorts. Second, there are many methods to determine baseline SCr; however, we did not fully discuss the effect of different baseline SCr definitions on the diagnosis of AKI. Further research is needed to investigate the influence of methods for estimating baseline SCr on pKDIGO. Third, although it has been suggested that the percentage change of SCr varies among children of different ages,^12^ it is unclear the exact trend of percentage change of SCr by age. In this manner, the criteria of percentage change of SCr were not revised in this study. Fourth, we compared the performance of AKI definitions using the urine output criteria only in the ICU cohort, because the urine output data was not acquired in the BCH cohort. Fifth, comparison of AKI definitions using different eGFR equations was only made in the BCH cohort, because the height information was quite rare in the ICU cohort.

## Conclusion

In summary, this study introduced a new pKDIGO definition of AKI for pediatric population. Validated with 2 retrospective cohorts, pKDIGO showed better ability to identify patients with AKI and predict in-hospital death risk. pKDIGO could be used to detect patients with AKI underdiagnosed using other definitions. Moreover, pKDIGO performed relatively stable in both general wards and ICUs, indicating that it can be used in a wide range of scenarios with mild or severe cases.

## Disclosure

All the authors declared no competing interests.

### Funding

This study was supported by 10.13039/501100001809National Natural Science Foundation of China (Grant No. 72174128); the Talent Development Plan for High-level Public Health Technical Personnel Project (Grant No. 2022-2-026); and 10.13039/501100005090Beijing Nova Program (Grant No. Z211100002121053). The findings and conclusions in this report are those of the authors and do not necessarily represent the official position of the funding institutions.

## Data Availability Statement

The data are available from the corresponding author on reasonable request.

## Author Contributions

XP led the whole study, critically revised the manuscript, and approved its final form. CZ and RY took part in the literature review and cleaning the raw dataset. XL, XN, and YP provided technological support in cleaning the raw dataset and validating the result. NZ was responsible for reviewing the diagnosis of AKI and the literature.

## Supplementary Material

Supplementary File (PDF) **Figure S1.** Standard eGFR and SCr by age. **Figure S2.** The distribution of relative changes and absolute changes (the modified AKI 1 criteria defined by pKDIGO) of Scr with age. **Figure S3.** The distribution of relative changes and absolute changes (the modified AKI 3 criteria defined by pKDIGO) of Scr with age. **Figure S4.** Distribution of eGFR of healthy children in China and the modified AKI stage 3 criteria defined by pKDIGO. **Figure S5.** Illustration of baseline SCr calculation. **Figure S6.** Study flow diagram of the general hospitalized cohort and ICU cohorts. **Figure S7.** Receiver operating characteristic curve of AKI definitions for predicting in-hospital death to examine the influence of different glomerular filtration rate equations. **Figure S8.** Receiver operating characteristic curve of AKI definitions for predicting in-hospital death to examine the influence of urine output. **Figure S9.** The mortality of different AKI stages diagnosed by pKDIGO vs KDIGO in BCH cohort, a Venn diagram. **Figure S10.** The mortality of different AKI stages diagnosed by pKDIGO vs KDIGO in ICU cohort, a Venn diagram. **Figure S11.** The mortality of different AKI stages diagnosed by pKDIGO vs. KDIGO vs. pRIFLE in BCH cohort for children younger than 6 months, a Venn diagram. **Figure S12.** The mortality of different AKI stages diagnosed by pKDIGO vs. KDIGO vs. pRIFLE in BCH cohort for children older than 6 months and younger than 2 years, a Venn diagram. **Figure S13.** The mortality of different AKI stages diagnosed by pKDIGO vs. KDIGO vs. pRIFLE in BCH cohort for children older than 2 years, a Venn diagram. **Table S1.** Other diagnostic criteria for AKI. **Table S2.** Outcomes in different patient groups. **Table S3.** Area under receiver operating characteristic curve of different definitions.

## Footnotes

Supplementary File (PDF)
**Figure S1.** Standard eGFR and SCr by age. **Figure S2.** The distribution of relative changes and absolute changes (the modified AKI 1 criteria defined by pKDIGO) of Scr with age. **Figure S3.** The distribution of relative changes and absolute changes (the modified AKI 3 criteria defined by pKDIGO) of Scr with age. **Figure S4.** Distribution of eGFR of healthy children in China and the modified AKI stage 3 criteria defined by pKDIGO. **Figure S5.** Illustration of baseline SCr calculation. **Figure S6.** Study flow diagram of the general hospitalized cohort and ICU cohorts. **Figure S7.** Receiver operating characteristic curve of AKI definitions for predicting in-hospital death to examine the influence of different glomerular filtration rate equations. **Figure S8.** Receiver operating characteristic curve of AKI definitions for predicting in-hospital death to examine the influence of urine output. **Figure S9.** The mortality of different AKI stages diagnosed by pKDIGO vs. KDIGO in BCH cohort, a Venn diagram. **Figure S10.** The mortality of different AKI stages diagnosed by pKDIGO vs. KDIGO in ICU cohort, a Venn diagram. **Figure S11.** The mortality of different AKI stages diagnosed by pKDIGO vs. KDIGO vs. pRIFLE in BCH cohort for children younger than 6 months, a Venn diagram. **Figure S12.** The mortality of different AKI stages diagnosed by pKDIGO vs. KDIGO vs. pRIFLE in BCH cohort for children older than 6 months and younger than 2 years, a Venn diagram. **Figure S13.** The mortality of different AKI stages diagnosed by pKDIGO vs. KDIGO vs. pRIFLE in BCH cohort for children older than 2 years, a Venn diagram. **Table S1.** Other diagnostic criteria for AKI. **Table S2.** Outcomes in different patient groups. **Table S3.** Area under receiver operating characteristic curve of different definitions.
