# Supplementary material for: Optimization of Kidney Disease: Improving Global Outcomes Criteria for AKI for Pediatric Population
Source: Kidney Int Rep. 2025 Nov 26;11(2):103701. doi: 10.1016/j.ekir.2025.11.026 (PMC12805013; doi:10.1016/j.ekir.2025.11.026)
Supplement: Supplementary File (PDF) — Figure S1. Standard eGFR and SCr by age. Figure S2. The distribution of relative changes and absolute changes (the modified AKI 1 criteria defined by pKDIGO) of Scr with age. Figure S3. The distribution of relative changes and absolute changes (the modified AKI 3 criteria defined by pKDIGO) of Scr with age. Figure S4. Distribution of eGFR of healthy children in China and the modified AKI stage 3 criteria defined by pKDIGO. Figure S5. Illustration of baseline SCr calculation. Figure S6. Study flow diagram of the general hospitalized cohort and ICU cohorts. Figure S7. Receiver operating characteristic curve of AKI definitions for predicting in-hospital death to examine the influence of different glomerular filtration rate equations. Figure S8. Receiver operating characteristic curve of AKI definitions for predicting in-hospital death to examine the influence of urine output. Figure S9. The mortality of different AKI stages diagnosed by pKDIGO vs KDIGO in BCH cohort, a Venn diagram. Figure S10. The mortality of different AKI stages diagnosed by pKDIGO vs KDIGO in ICU cohort, a Venn diagram. Figure S11. The mortality of different AKI stages diagnosed by pKDIGO vs. KDIGO vs. pRIFLE in BCH cohort for children younger than 6 months, a Venn diagram. Figure S12. The mortality of different AKI stages diagnosed by pKDIGO vs. KDIGO vs. pRIFLE in BCH cohort for children older than 6 months and younger than 2 years, a Venn diagram. Figure S13. The mortality of different AKI stages diagnosed by pKDIGO vs. KDIGO vs. pRIFLE in BCH cohort for children older than 2 years, a Venn diagram. Table S1. Other diagnostic criteria for AKI. Table S2. Outcomes in different patient groups. Table S3. Area under receiver operating characteristic curve of different definitions. [file mmc1.pdf]

Optimization of KDIGO criteria for AKI for pediatric population

Chao Zhang[1#], Ruohua Yan[1#], Xiaohang Liu[1], Xiaolu Nie[1], Yaguang Peng[1], Nan Zhou[2], Xiaoxia Peng[1]

1 Center for Clinical Epidemiology and Evidence-based Medicine, Beijing Children's Hospital, Capital Medical University, National Center for Children Health, Beijing, China

2 Department of Nephrology, Beijing Children's Hospital, Capital Medical University, National Center for Children Health, Beijing, China

# There authors contributed equally to this work.

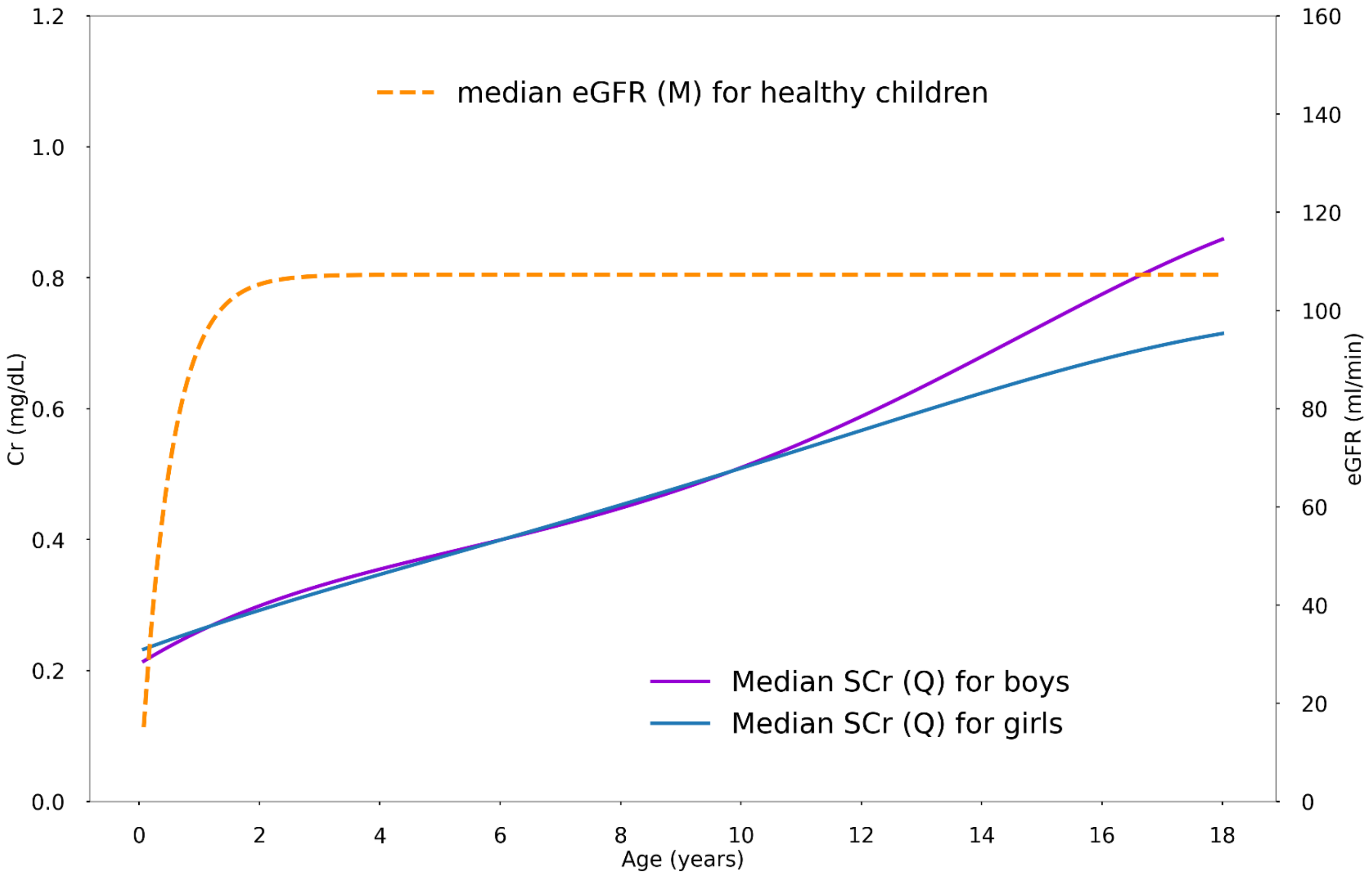

Figure S1: Median eGFR (M) and median SCr (Q) by age.

Abbreviations: eGFR: estimated glomerular filtration rate; SCr: serum creatinine.

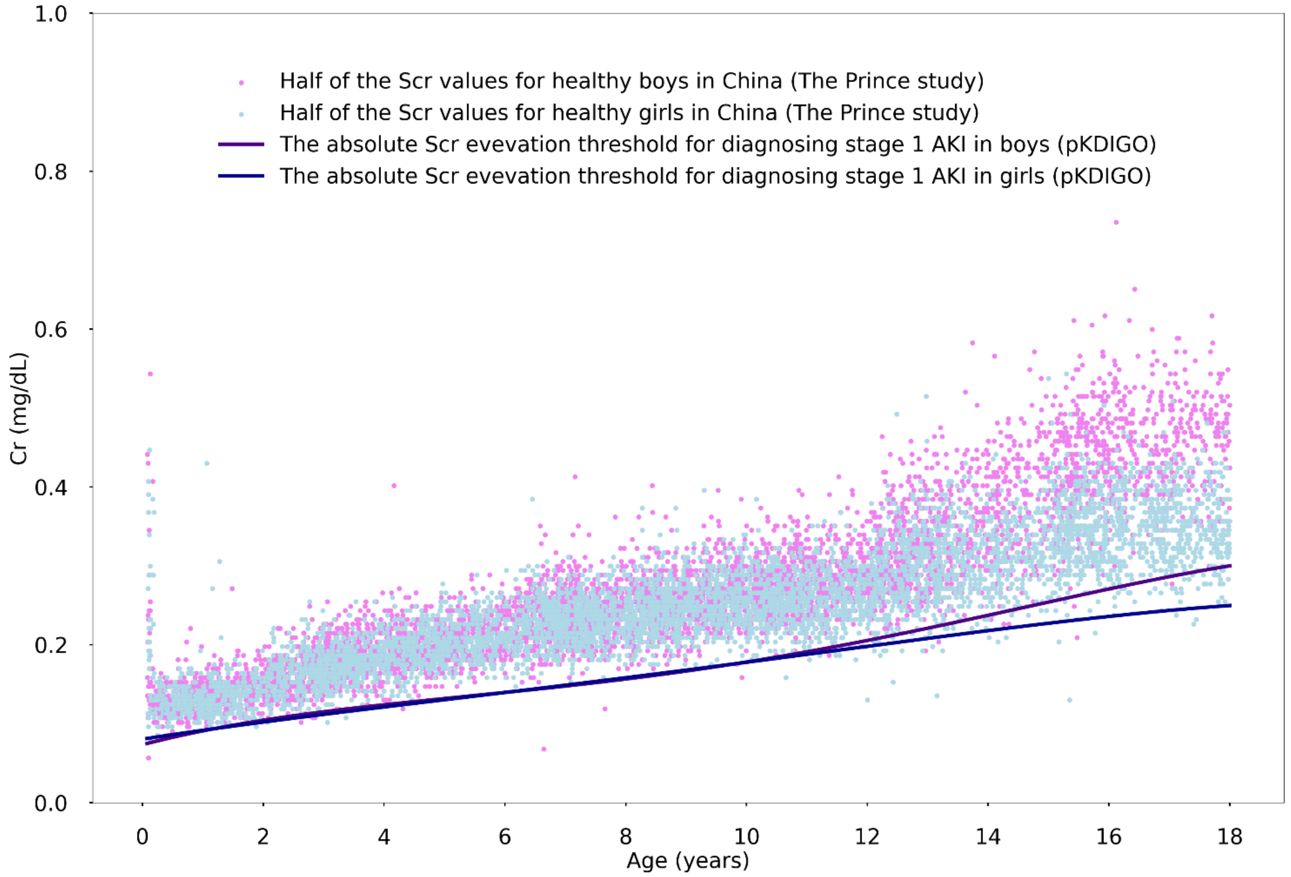

Figure S2: The distribution of relative changes and absolute changes (the modified AKI 1 criteria defined by pKDIGO) of Scr with age.

Abbreviations: PRINCE: The Pediatric Reference **Intervals** in China study; SCr: serum creatinine; pKDIGO: pediatric version of KDIGO.

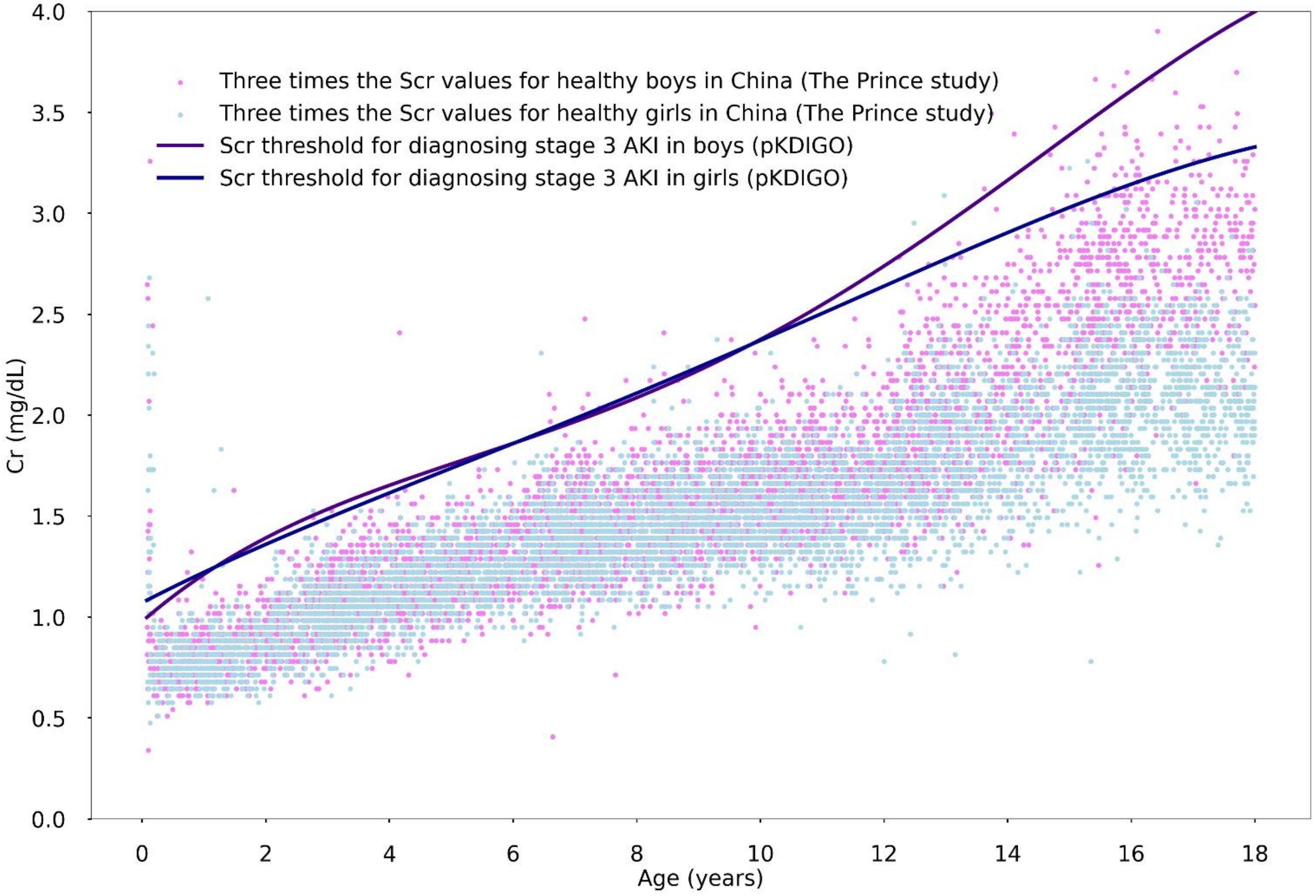

Figure S3: The distribution of relative changes and absolute changes (the modified AKI 3 criteria defined by pKDIGO) of Scr with age.

Abbreviations: PRINCE: The Pediatric Reference Intervals in China study; SCr: serum creatinine; pKDIGO: pediatric version of KDIGO.

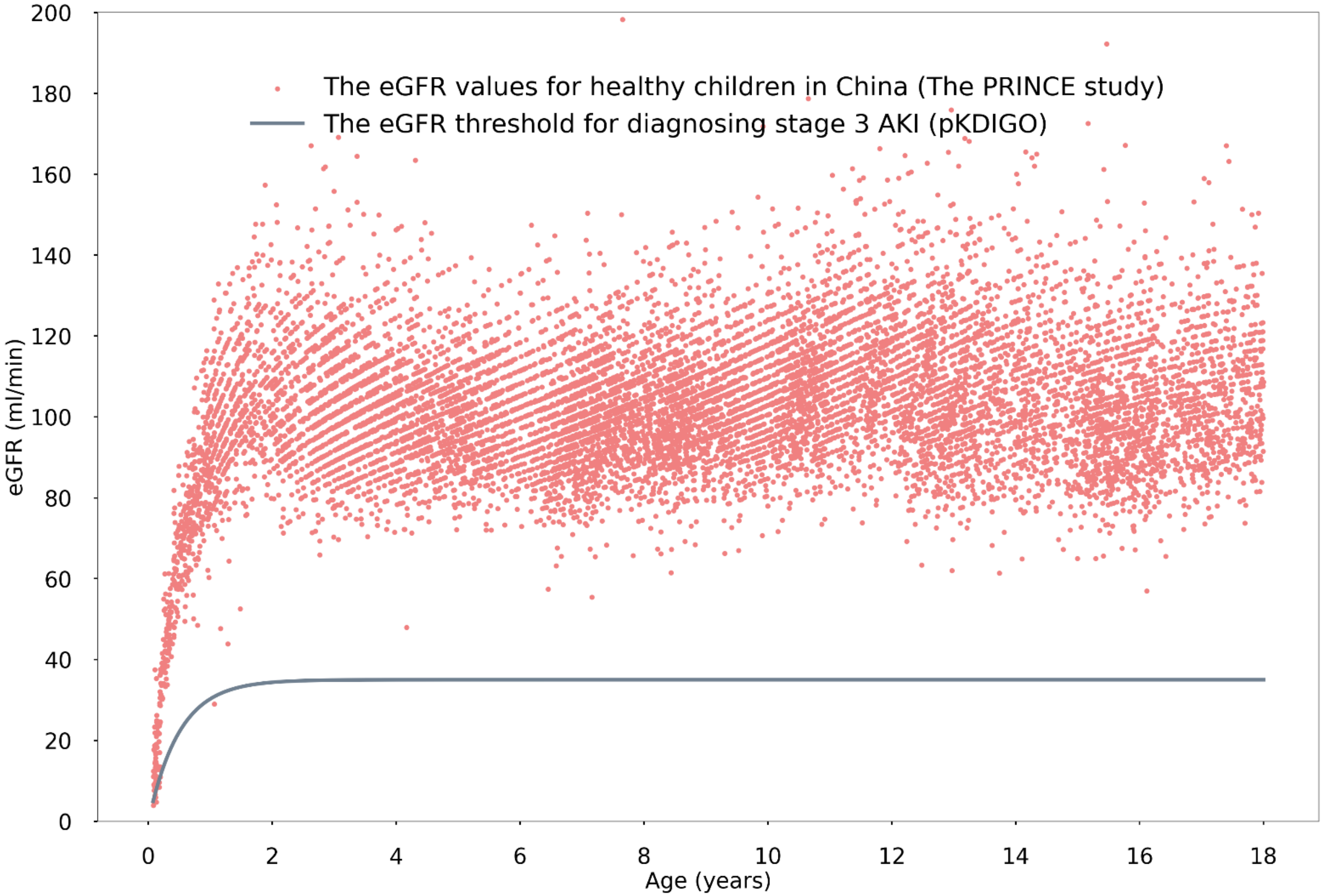

Figure S4: Distribution of eGFR of healthy children in China and the modified AKI stage 3 criteria defined by pKDIGO.

Abbreviations: eGFR: estimated Glomerular Filtration rate; PRINCE: The Pediatric Reference Intervals in China study; pKDIGO: pediatric version of KDIGO.

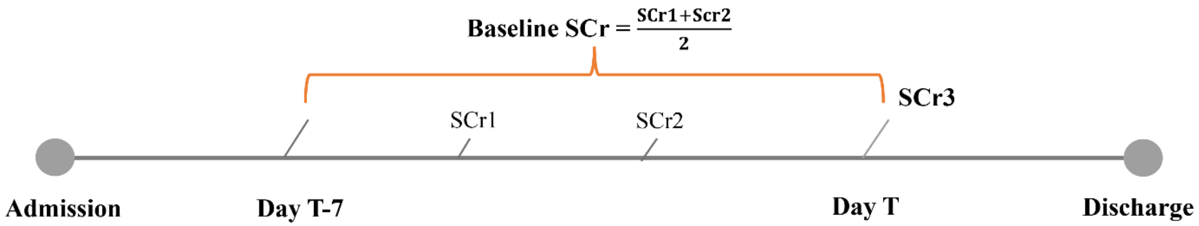

Figure S5: Illustration of baseline SCr calculation.

Abbreviations: SCr: Serum Creatinine.

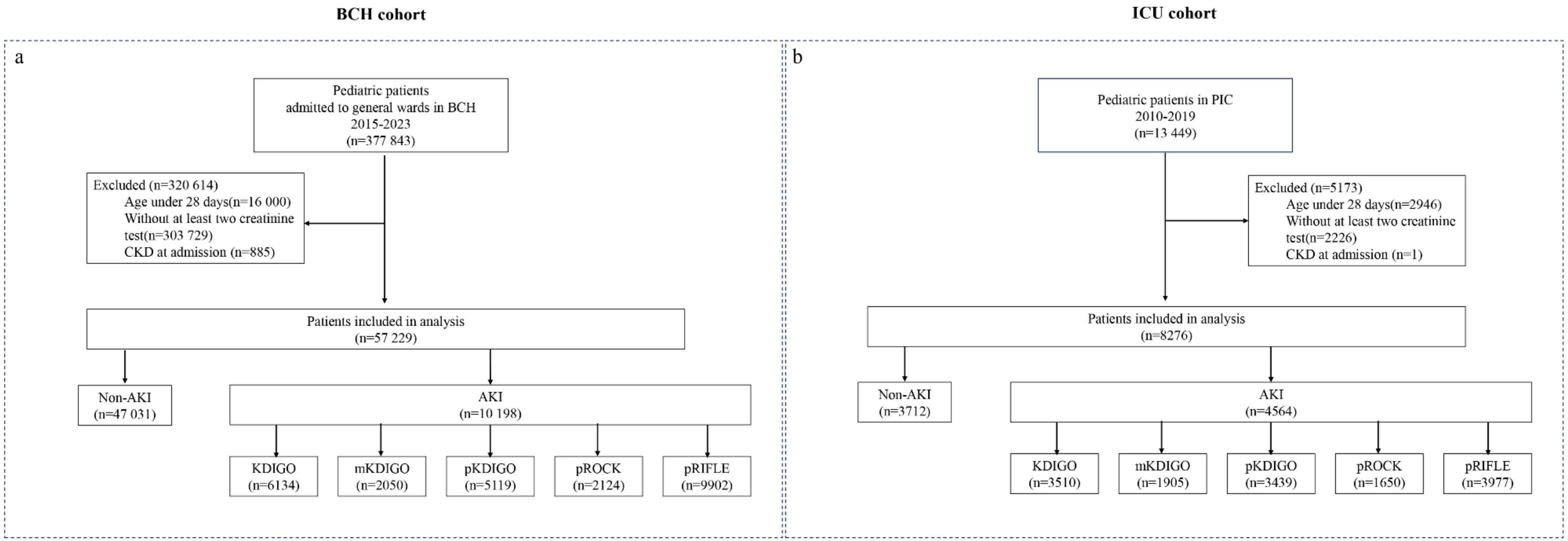

Figure S6: Study flow diagram of the general hospitalized cohort and ICU cohorts.

(a) BCH cohort. (b) ICU cohort.

Abbreviations: AKI: acute kidney injury; BCH: Beijing Children's Hospital; CKD: chronic kidney disease; ICU: intensive care unit; KDIGO: Kidney Diseases Improving Global Outcomes; mKDIGO: modified KDIGO; PIC: pediatric intensive care; pKDIGO: pediatric version of KDIGO; pROCK: pediatric reference change value optimized for AKI in children; pRIFLE: pediatric RIFLE.

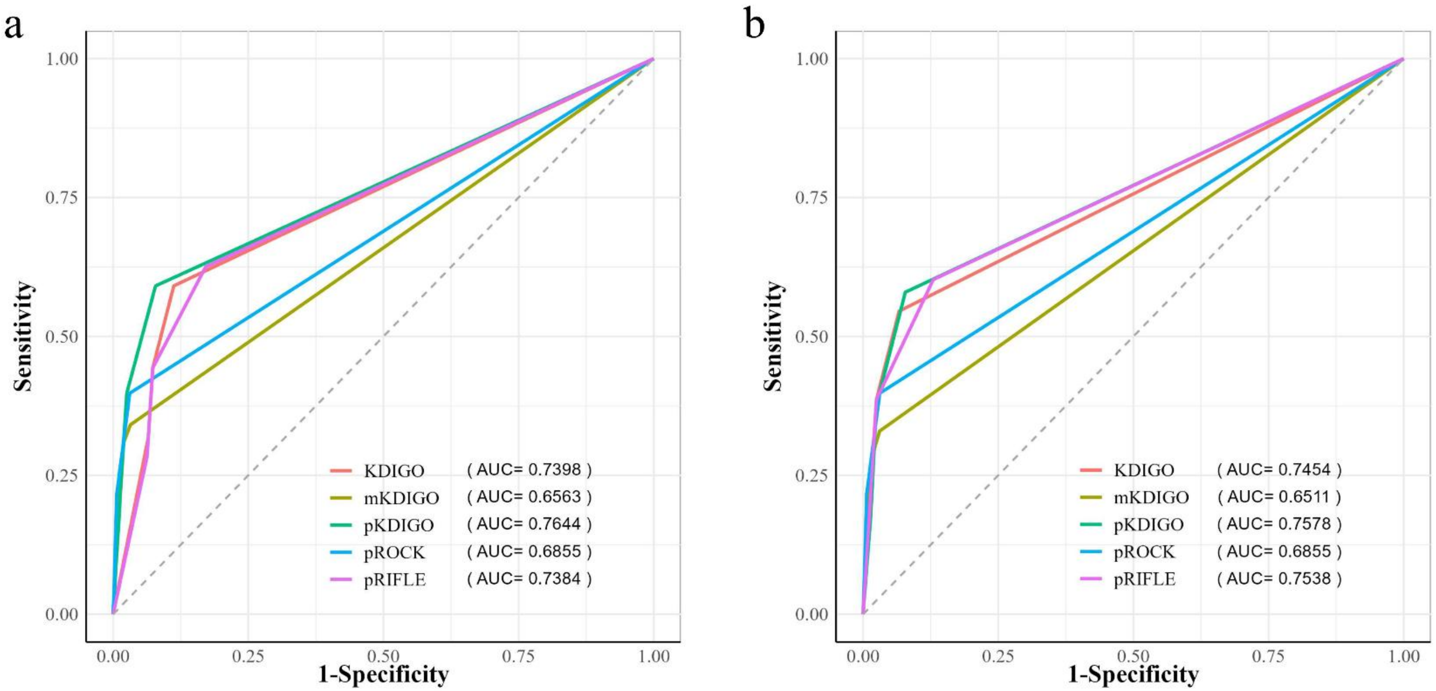

Figure S7: Receiver operating characteristic curve of AKI definitions for predicting in-hospital death to examine the influence of different glomerular filtration rate equations.

(a) FAS equation. (b) Schwartz equation.

Abbreviations: KDIGO: Kidney Diseases Improving Global Outcomes; mKDIGO: modified KDIGO; pKDIGO: pediatric version of KDIGO; pROCK: pediatric reference change value optimized for AKI in children; pRIFLE: pediatric RIFLE.

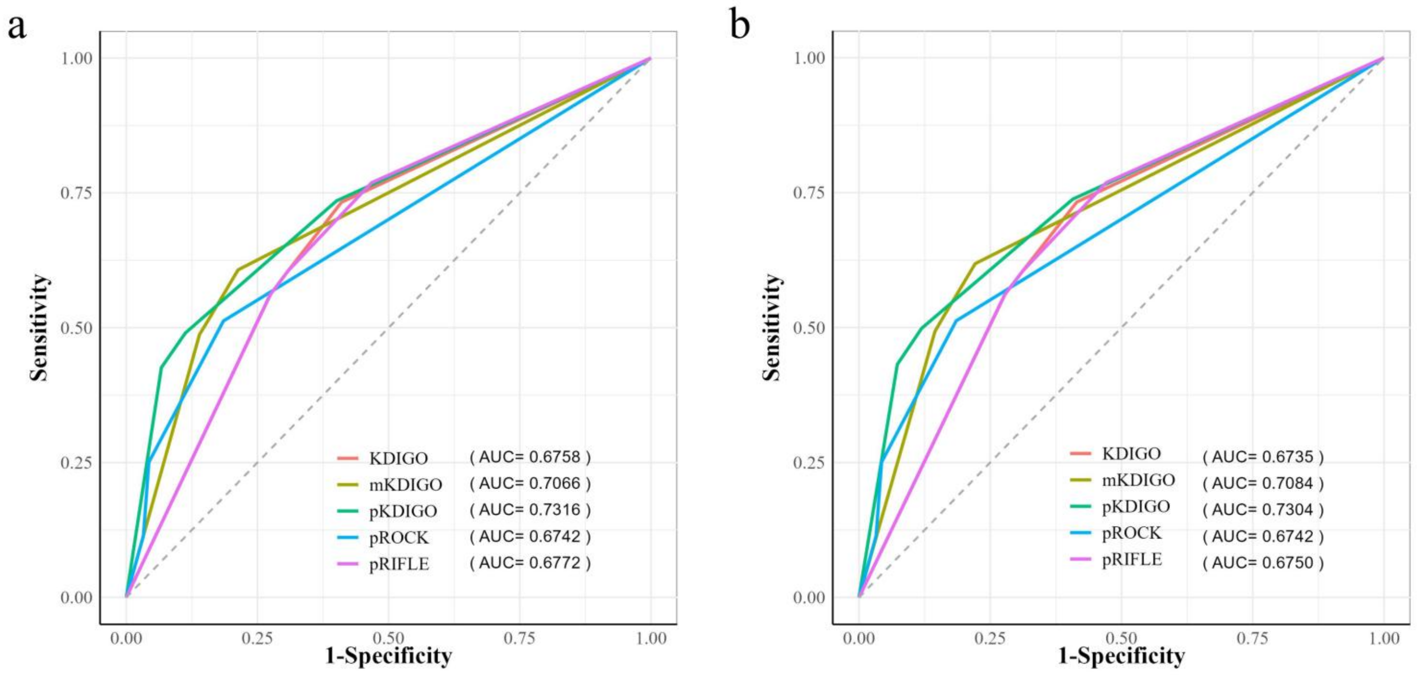

Figure S8: Receiver operating characteristic curve of AKI definitions for predicting in-hospital death to examine the influence of urine output.

(a) without urine output. (b) include urine output.

Abbreviations: KDIGO: Kidney Diseases Improving Global Outcomes; mKDIGO: modified KDIGO; pKDIGO: pediatric version of KDIGO; pROCK: pediatric reference change value optimized for AKI in children; pRIFLE: pediatric RIFLE.

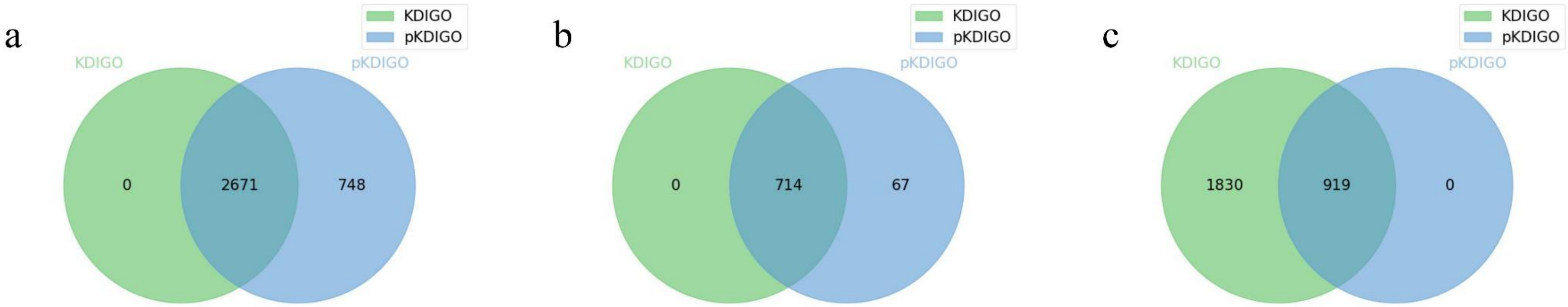

Number of cases	Mortality, n(%)
0	NA
2671	56 (2.10)
748	18 (2.41)

Number of cases	Mortality, n(%)
0	NA
714	27 (3.78)
67	4 (5.97)

Number of cases	Mortality, n(%)
1830	13 (0.71)
919	43 (4.68)
0	NA

Figure S9: The mortality of different AKI stages diagnosed by KDIGO vs pKDIGO in BCH cohort, a Venn diagram.
Population size and mortality rate is shown in different groups.
(a) Stage 1 AKI. (b) Stage 2 AKI. (c) Stage 3 AKI.
Abbreviations: BCH: Beijing Children's Hospital; KDIGO: Kidney Diseases Improving Global Outcomes; pKDIGO: pediatric version of KDIGO.

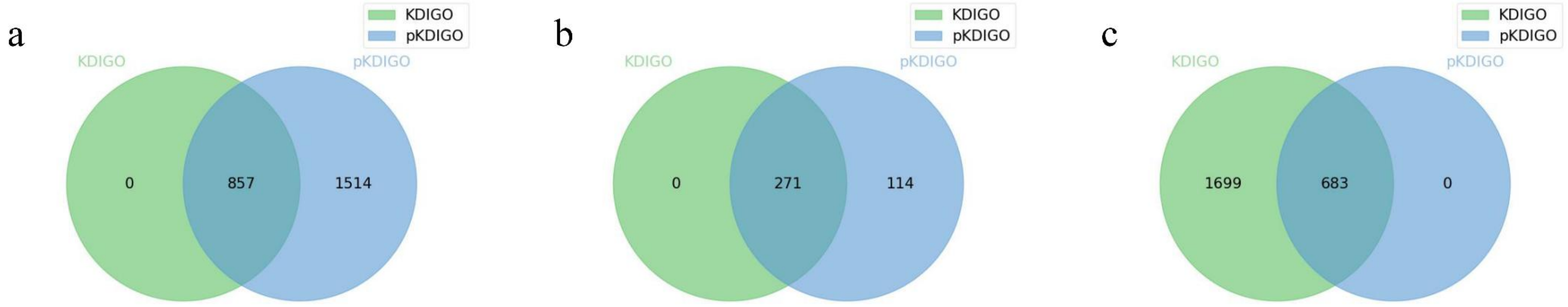

(a)

Number of cases	Mortality, n(%)
0	NA
857	46 (5.37)
1514	42 (2.77)

(b)

Number of cases	Mortality, n(%)
0	NA
271	15 (5.54)
114	8 (7.02)

(c)

Number of cases	Mortality, n(%)
1699	49 (2.88)
683	153 (22.4)
0	NA

Figure S10: The mortality of different AKI stages diagnosed by KDIGO vs pKDIGO in ICU cohort, a Venn diagram.
Population size and mortality rate is shown in different groups.
(a) Stage 1 AKI. (b) Stage 2 AKI. (c) Stage 3 AKI.
Abbreviations: ICU: Intensive Care Unit; KDIGO: Kidney Diseases Improving Global Outcomes; pKDIGO: pediatric version of KDIGO.

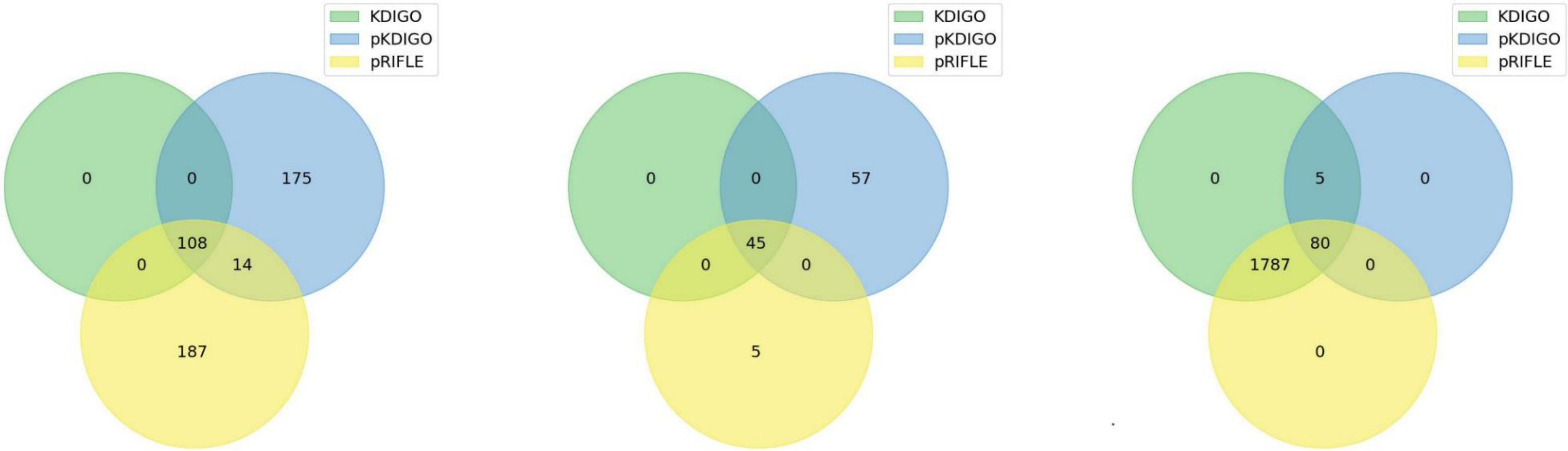

	Number of cases	Mortality, n(%)
KDIGO	108	3 (2.78)
pKDIGO	297	7 (2.36)
pRIFLE	309	6 (1.94)

	Number of cases	Mortality, n(%)
KDIGO	45	3 (6.67)
pKDIGO	102	4 (3.92)
pRIFLE	50	3 (6.00)

	Number of cases	Mortality, n(%)
KDIGO	1872	17 (0.91)
pKDIGO	85	8 (9.41)
pRIFLE	1867	17 (0.91)

Figure S11: The mortality of different AKI stages diagnosed by KDIGO vs. pKDIGO vs. pRIFLE in BCH cohort for children younger than six months, a Venn diagram.

Population size and mortality rate is shown in different groups.

(a) KDIGO. (b) pKDIGO. (c) pRIFLE.

Abbreviations: ICU: Intensive Care Unit; KDIGO: Kidney Diseases Improving Global Outcomes; pKDIGO: pediatric version of KDIGO; pRIFLE: pediatric RIFLE.

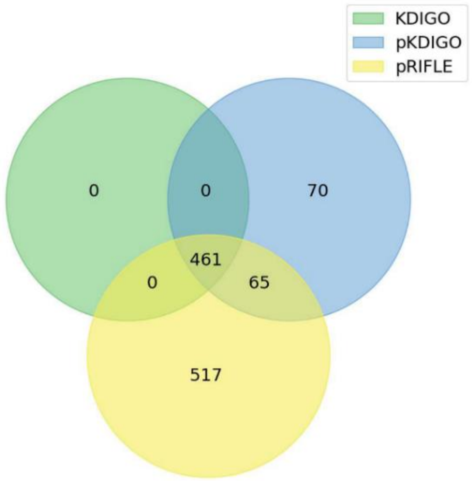

	Number of cases	Mortality, n(%)
KDIGO	461	17 (3.69)
pKDIGO	596	23 (3.86)
pRIFLE	1043	22 (2.11)

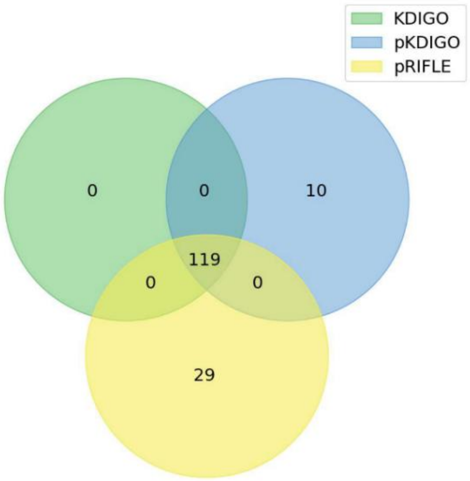

	Number of cases	Mortality, n(%)
KDIGO	119	8 (6.72)
pKDIGO	129	11 (8.52)
pRIFLE	148	10 (6.76)

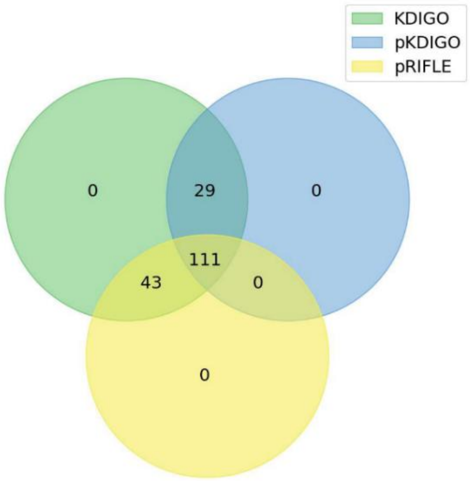

	Number of cases	Mortality, n(%)
KDIGO	183	18 (9.84)
pKDIGO	140	14 (10.00)
pRIFLE	154	16 (10.39)

Figure S12: The mortality of different AKI stages diagnosed by KDIGO vs. pKDIGO vs. pRIFLE in BCH cohort for children older than six months and younger than 2 years, a Venn diagram.

Population size and mortality rate is shown in different groups.

(a) KDIGO. (b) pKDIGO. (c) pRIFLE.

Abbreviations: ICU: Intensive Care Unit; KDIGO: Kidney Diseases Improving Global Outcomes; pKDIGO: pediatric version of KDIGO; pRIFLE: pediatric RIFLE.

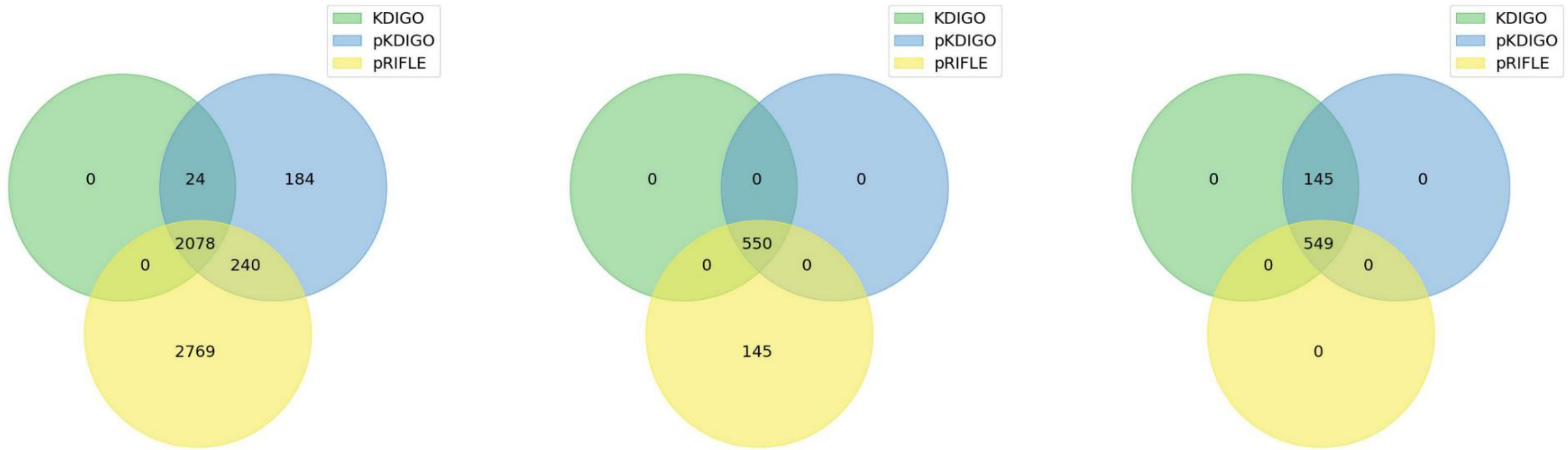

	Number of cases	Mortality, n(%)
KDIGO	2102	36 (1.71)
pKDIGO	2526	44 (1.74)
pRIFLE	5087	43 (0.85)

	Number of cases	Mortality, n(%)
KDIGO	550	16 (2.91)
pKDIGO	550	16 (2.91)
pRIFLE	695	22 (3.17)

	Number of cases	Mortality, n(%)
KDIGO	694	21 (3.03)
pKDIGO	694	21 (3.03)
pRIFLE	549	15 (2.73)

Figure S13: The mortality of different AKI stages diagnosed by pKDIGO vs. KDIGO vs. pRIFLE in BCH cohort for children older than 2 years, a Venn diagram.

Population size and mortality rate is shown in different groups.

(a) KDIGO. (b) pKDIGO. (c) pRIFLE.

Abbreviations: ICU: Intensive Care Unit; KDIGO: Kidney Diseases Improving Global Outcomes; pKDIGO: pediatric version of KDIGO; pRIFLE: pediatric RIFLE.

Table S1. Other diagnostic criteria for AKI

	Stage	SCr	eGFR	Urine output
**mKDIGO**	1	increase 50–99 %[b] from baseline or rise of 0.3 mg/dL[a], and SCr ⩾0.5 mg/dL		＜0.5 mL/kg/h for 6 h
	2	increase 100–199 %[b] from baseline, and SCr ⩾0.5 mg/dL		＜0.5 mL/kg/h for 12 h
	3	increase ⩾200 %[b] from baseline or SCr ⩾4 mg/dL, and SCr ⩾ 0.5 mg/dL	eGFR ＜35 ml/min/1.73 m^2	＜0.3 mL/kg/h for 24 h or anuria for 12 h
**pROCK**	1	increase 30–59 %[b] and absolute SCr increase 0.2-0.4 mg/dL[b]		
	2	increase 60–119 %[b] and absolute SCr increase 0.5-0.9 mg/dL[b]		
	3	increase ⩾120 %[b] and absolute SCr increase 1 mg/dL[b]		
**pRIFLE**	1		decrease 25–49 %[b]	＜0.5 mL/kg/h for 6 h
	2		decrease 50–74 %[b]	＜0.5 mL/kg/h for 12 h
	3		decrease 75 %[b] or eGFR ＜35 ml/min/1.73 m^2	＜0.3 mL/kg/h for 24 h or anuria for 12 h

[a] increase within 48 h
[b] increase within 7 days
Abbreviations: SCr: serum creatinine; eGFR: estimated glomerular filtration rate; mKDIGO: modified KDIGO; pROCK: pediatric reference change value optimized for AKI in children; pRIFLE: pediatric RIFLE.

Table S2. Outcomes in different patient groups

Groups	Total	Death, n (%)
All children in BCH cohort	57229	259 (0.45%)
No AKI	47031	96 (0.20%)
AKI cases diagnosed by KDIGO	6134	139 (2.27 %)
AKI cases diagnosed by mKDIGO	2050	78 (3.80 %)
AKI cases diagnosed by pKDIGO	5119	148 (2.89 %)
AKI cases diagnosed by pROCK	2124	83 (3.91 %)
AKI cases diagnosed by pRIFLE	9902	154 (1.56 %)
AKI cases diagnosed solely through KDIGO and missed by others	0	NA
AKI cases diagnosed solely through mKDIGO and missed by others	0	NA
AKI cases diagnosed solely through pKDIGO and missed by others	245	9 (3.67 %)
AKI cases diagnosed solely through pROCK and missed by others	22	0 (0 %)
AKI cases diagnosed solely through pRIFLE and missed by others	3333	9 (0.27 %)
All children in ICU cohort	8276	359 (4.34 %)
No AKI	3712	74 (1.99 %)
AKI cases diagnosed by KDIGO	3510	263 (7.49 %)
AKI cases diagnosed by mKDIGO	1905	218 (11.44 %)
AKI cases diagnosed by pKDIGO	3439	264 (7.68 %)
AKI cases diagnosed by pROCK	1650	184 (11.15 %)
AKI cases diagnosed by pRIFLE	3977	276 (6.94 %)
AKI cases diagnosed solely through KDIGO and missed by others	0	NA
AKI cases diagnosed solely through mKDIGO and missed by others	0	NA
AKI cases diagnosed solely through pKDIGO and missed by others	575	7 (1.22 %)
AKI cases diagnosed solely through pROCK and missed by others	1	0 (0 %)
AKI cases diagnosed solely through pRIFLE and missed by others	132	1 (0.76 %)

Abbreviations: AKI: acute kidney injury; BCH: Beijing Children's Hospital; ICU: intensive care unit; KDIGO: Kidney Diseases Improving Global Outcomes; mKDIGO: modified KDIGO; pKDIGO: pediatric version of KDIGO; pROCK: pediatric reference change value optimized for AKI in children; pRIFLE: pediatric RIFLE.

Table S3. Area under receiver operating characteristic curve of different definitions

Definitions	**BCH cohort**	**ICU cohort**
KDIGO	0.72 (0.68, 0.75)	0.68 (0.65, 0.70)
mKDIGO	0.63 (0.61, 0.66)	0.71 (0.68, 0.73)
pKDIGO	0.75 (0.72, 0.78)	0.73 (0.70, 0.76)
pROCK	0.64 (0.61, 0.67)	0.67 (0.65, 0.70)
pRIFLE	0.72 (0.69, 0.75)	0.68 (0.65, 0.70)

Abbreviations: BCH: Beijing Children's Hospital; ICU: intensive care unit; CI: confidence interval; KDIGO: Kidney Diseases Improving Global Outcomes; mKDIGO: modified KDIGO; pKDIGO: pediatric version of KDIGO; pROCK: pediatric reference change value optimized for AKI in children; pRIFLE: pediatric RIFLE; PLOS: prolonged length of stay.
